# Reconstruction of the Shallow Acetabulum With a Combination of Autologous Bulk and Impaction Bone Grafting Fixed by Cement

**DOI:** 10.1007/s11999-016-5107-6

**Published:** 2016-11-11

**Authors:** Masaaki Maruyama, Shinji Wakabayashi, Hiroshi Ota, Keiji Tensho

**Affiliations:** 1 Department of Orthopedic Surgery, Shinonoi General Hospital, 666-1 Ai, Shinonoi, Nagano 388-8004 Japan; 2 Department of Orthopedic Surgery, Chushin Mastumoto Hospital, Matsumoto Medical Center, Matsumoto, Nagano Japan; 3 Department of Orthopedic Surgery, Kokuho Yodakubo Hospital, Nagawa, Nagano Japan; 4 Department of Orthopedic Surgery, Shinshu University School of Medicine, Matsumoto, Nagano Japan

## Abstract

**Background:**

Acetabular bone deficiency, especially proximal and lateral deficiency, is a difficult technical problem during primary total hip arthroplasty (THA) in developmental dysplasia of the hip (DDH). We report a new reconstruction method using a medial-reduced cemented socket and additional bulk bone in conjunction with impaction morselized bone grafting (additional bulk bone grafting method).

**Questions/purposes:**

In a population of patients with acetabular dysplasia undergoing THA using a medial-reduced cemented socket and additional bulk bone with impacted morselized bone grafting, we evaluated (1) the radiographic appearance of bone graft; (2) the proportion of cups that developed loosening and subsequent revision; and (3) clinical results (outcome scores and complications).

**Methods:**

Forty percent of 330 THAs for DDH performed at one center between 1999 and 2009 were defined as shallow dysplastic hips. The additional bulk bone grafting method was performed on 102 THAs with shallow acetabulum (31% for DDH) at one center between 1999 and 2009. We used this approach and technique for shallow acetabuli when a cup protruded from the lateral acetabular edge in preoperative templating. The other 132 dysplastic hips without bone grafting had THA performed at the same periods and served as a control. Acetabuli were defined as shallow when the depth was less than or equal to one-fifth of the pelvic height (cranial-caudal length on radiograph). The additional bulk bone grafting technique was as follows: the resected femoral head was sectioned at 1 to 2 cm thickness, and a suitable size of the bulk bone graft was placed on the lateral iliac cortex and fixed by poly-l-lactate absorbable screws. Autologous impaction morselized bone grafting, with or without hydroxyapatite granules, was performed along with the implantation of a medial-reduced cemented socket. We defined an “incorporated” graft as remodeling and trabeculation including rounding off of the protruding edge of a graft beyond the socket. Radiographic criteria used for determining loosening were migration or a continuous radiolucent zone between the prosthesis/bone cement and host bone. Clinical outcomes were assessed using the Japanese Orthopaedic Association (JOA) and the Merle d’Aubigne and Postel score; complications were tallied from chart review. The followup was 10 ± 3 years (range, 6–15 years).

**Results:**

One acetabular component (1%) with severe shallow and steep acetabuli showed definite radiographic evidence of loosening and was revised. Clinically, the mean JOA score for the hips treated with additional bulk bone grafting THA in this study improved from 39 ± 10 points preoperatively to 95 ± 5 points postoperatively (p < 0.05, paired t-test). The mean Merle d’Aubigne and Postel score for the hips improved from 7 ± 2 points to 17 ± 1 points (p < 0.05, paired t-test). Complications included a Trendelenburg sign in one hip, dislocation in one, and transient partial sciatic nerve palsy in one. Within 3 years 6 months postoperatively, 101 of 102 additional bulk bone grafting cases showed successful bone remodeling and bone graft reorientation without collapse on radiographs. Partial resorption of the additional bone graft on the lateral side was observed in two hips (2%) with socket abduction angles of < 35°.

**Conclusions:**

Achieving stable acetabular fixation is often challenging in the dysplastic hip, especially shallow acetabulum, and a variety of techniques have been described. Early results of combining bulk graft with impaction of morselized graft are promising. Although each surgical technique was well established, further investigation for clinical results of a combination of these techniques might be necessary to confirm longer term outcomes.

**Level of Evidence:**

Level IV, therapeutic study.

## Introduction

Developmental dysplasia of the hip (DDH), including acetabular dysplasia, subluxation, or dislocation, is relatively common, eg, 1.7% to 10% [2, 3, 12, 22, 27, 30]. Yoshimura et al. [41] reported that the dimensions of dysplastic acetabuli are considerably shallower in Japanese subjects than in their British counterparts of similar age (60–79 years) and sex. Total hip arthroplasty (THA) in patients with DDH is technically demanding because of the altered hip anatomy. For acetabular reconstruction, the following techniques are described: (1) cranial positioning of the acetabular component (high hip center for steep acetabuli) [17, 31]; (2) medial protrusion technique (cotyloplasty for shallow acetabuli) [8, 19]; (3) customized acetabular augments (three-dimensional printing) [4] or high-porosity sockets such as Trabecular Metal, Regenerex, or Tritanium; (4) acetabular roof reconstruction with autologous bone grafting [33]; and (5) graft augmentation by rim mesh or hooked reinforcement ring devices with impaction morselized bone grafting [1, 15, 35]. In the first three techniques, however, acetabular bone stock is not preserved and the original hip center may be displaced. In addition, it is important to preserve and increase bone stock in primary THA, because the most difficult problems in revision hip arthroplasty are large bone deficiency and its reconstruction.

In this study, we focused on reconstruction of a shallow/steep acetabulum with minimum bulk bone coverage with the goal of placing an acetabular component at the anatomic position with adequate medialization. This reconstruction included: (1) medialization of the hip center by using a medial-reduced socket [25]; and (2) increase of transverse bone coverage by using additional bulk bone grafting techniques instead of interpositional bulk bone grafting techniques. The morselized bone grafting technique is a well-established method for reconstruction of bone defects [15, 33], but it is difficult to spread the transverse diameter of the acetabulum. Therefore, we combined this grafting technique with additional bulk bone grafting in this series.

In a population of patients with acetabular dysplasia undergoing THA using a medial-reduced cemented socket with a concentric center and additional bulk bone with impacted morselized bone grafting, we evaluated (1) the radiographic appearance of bone graft; (2) the proportion of cups that developed loosening and subsequent revision; and (3) clinical results (outcome scores and complications). We present the results of clinical outcomes and graft integrity, as measured by radiographs, maintained at latest followup.

## Patients and Methods

**Fig. 1:**
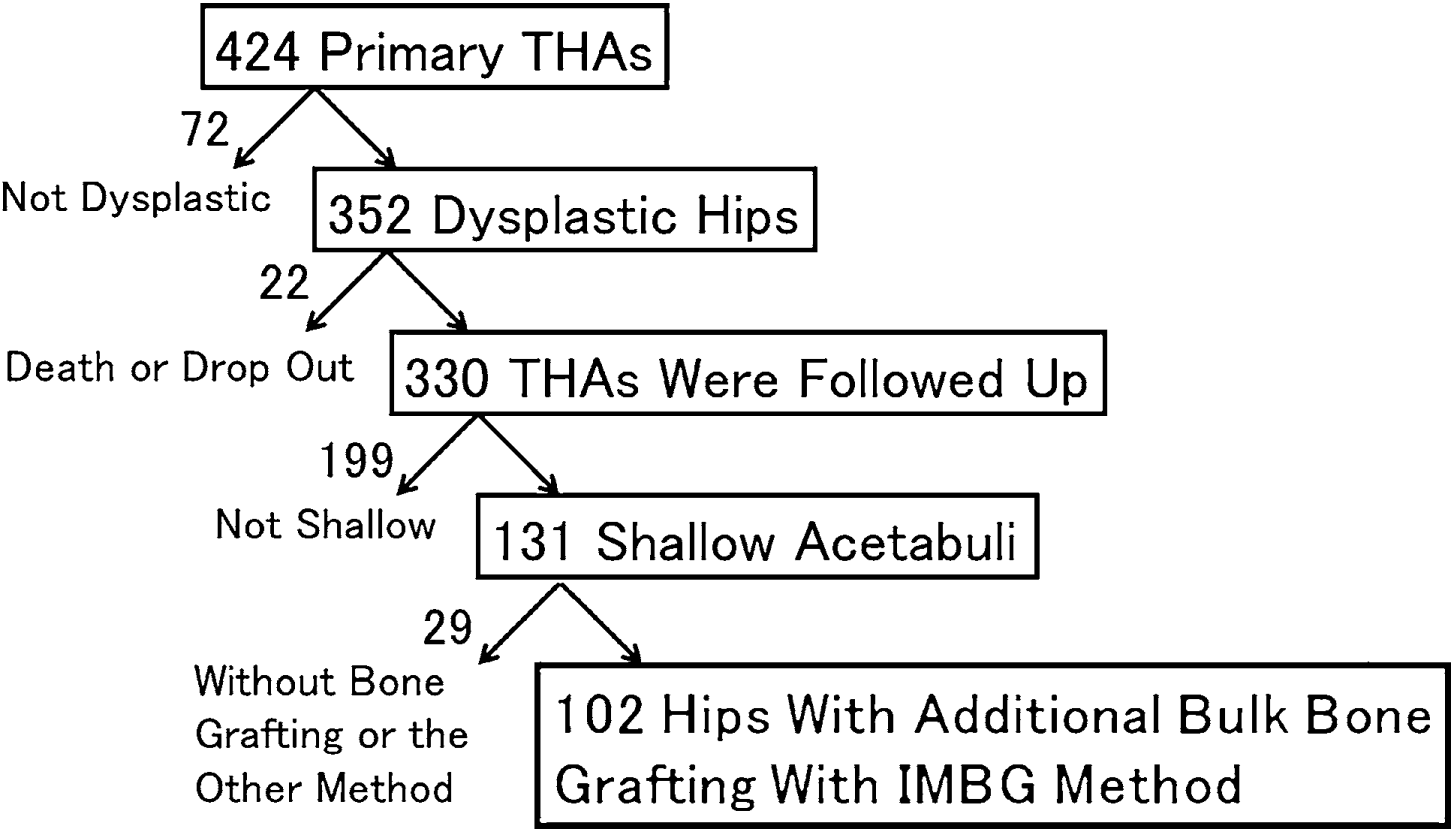
Flowchart shows the study patients with THA. IMBG = impaction morselized bone grafting.

The study was approved by the institutional ethical committee. Between October 29, 1999, and April 30, 2009, we performed 424 primary THAs using highly crosslinked polyethylene cemented sockets combined with zirconia or alumina heads at our hospital. Of those hips, 301 patients (352 hips [83%]) were diagnosed with DDH before surgery. Within 6 years postoperatively, 10 patients (10 hips) were excluded from the study because of death from unrelated causes. We lost track of 10 patients (12 hips) before the minimum long-term postoperative followup of 6 years. We followed up 281 patients (330 hips with dysplasia) who underwent THA. Of 330 dysplastic hips, we used a medial-reduced cemented socket with concentric center and additional bulk bone with impacted morselized bone grafting for 102 shallow acetabuli when an appropriate-sized cup protruded from the lateral acetabular edge at preoperative templating (Fig. 1). The cup size was commonly one-fifth of the pelvic height.

**Fig. 2:**
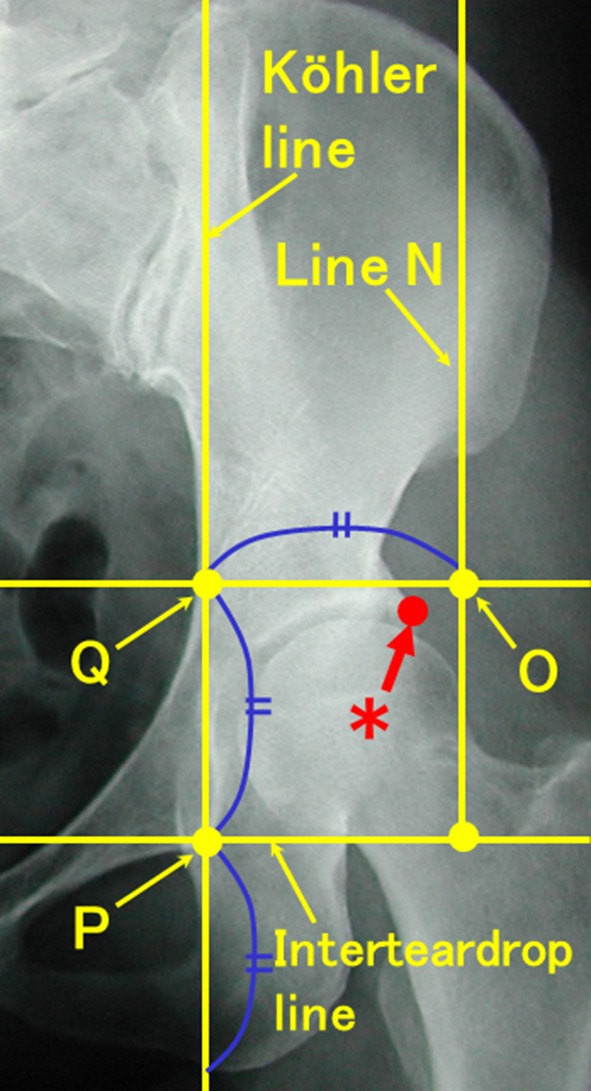
Radiogram showing shallow dysplastic hip. Shallow acetabulum was defined as that of the lateral acetabular edge medial to line N. PQ = a length of one-fifth of the pelvic height, which is defined as a length along the Köhler line from a line connecting bilateral ischial tuberosities to a line connecting bilateral iliac crests; QO = a line parallel to a line connecting bilateral teardrops (interteardrop line), PQ = QO; Line N = a line parallel to the Köhler line through point “O.” ^*^Lateral acetabular edge without osteophyte.

According to our criteria, 40% (131 hips) of 330 hips were classified with shallow acetabuli. Shallow acetabulum was defined as that of the lateral acetabular edge without osteophyte located medial to line N (Fig. 2). Line N was a line parallel to the Köhler line and through the point that was located laterally a length of one-fifth of the pelvic height along the teardrop line from the point two-fifths of the pelvic height (cranial-caudal length on radiograph) on the line parallel to the Köhler line through the acetabular floor.

These 102 hips consisted of 85 female (99 hips) and three male (three patients) including 10 hips with Crowe [7] Type III and three hips with Crowe Type IV (12 women and one man). At the time of surgery, the average patient age was 58 ± 10 years (range, 34–86 years). Patient mean body height was 153 ± 6 cm, mean body weight was 54 ± 11 kg, and mean body mass index was 23 ± 4 kg/m^2^. The mean followup was 10 ± 3 years (range, 6–15 years). The other 132 dysplastic hips without bone grafting THA performed during the same periods served as a control (Table 1).

**Table 1:** Demographic data and results of the study patients undergoing THA

Variable	Ad-BBG patients (n = 102 hips)	No BG patients (n = 132 hips)	t-test
Age at THA (years)	58 ± 10	65 ± 9	p < 0.05
Body height (cm)	153 ± 6	153 ± 7	NS
Body weight (kg)	55 ± 11	56 ± 10	NS
Body mass index (kg/m^2^)	23 ± 4	24 ± 4	NS
Operation time (minutes)	154 ± 41	116 ± 30	p < 0.05
Abduction angle of the socket (degrees)	42 ± 5	44 ± 5	p < 0.05
JOA score (preoperative)	39 ± 10	41 ± 11	NS
M&D score (preoperative)	7 ± 2	7 ± 1	NS
JOA score (at final followup)	95 ± 5	94 ± 5	NS
M&D score (at final followup)	17 ± 1	17 ± 1	NS

Values are mean ± SD; Ad-BBG = additional bulk bone graft in cases with impaction morselized cancellous bone grafting; BG = bone grafting; JOA score = Japanese Orthopaedic Association hip score; M&P score = Merle d’Aubigné-Postel score; NS = nonsignificant.

**Fig. 3:**
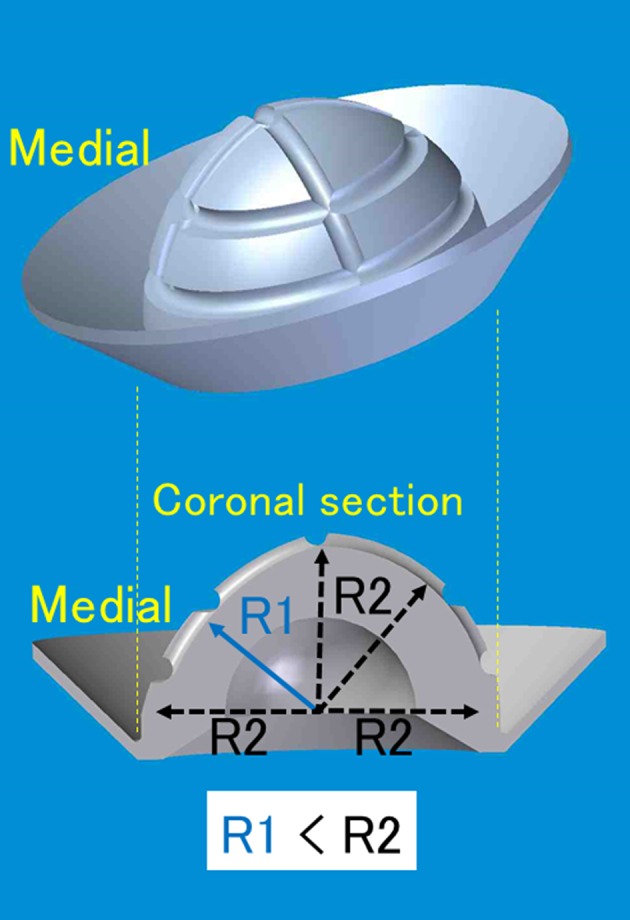
The 2-mm medial-reduced flange socket (FL-R socket) with concentric center is shown. This socket is different from the Charnley offset-bore acetabular cup with an eccentric center [16].

A new type of artificial acetabular socket, with 2-mm reduction of the medial polyethylene thickness, was clinically introduced to medialize the hip center and decrease the coverage ratio of the bulk bone graft. We noted that the shape of the medial part of the socket should not be spheroid, because the acetabular floor is relatively flat compared with the whole acetabulum. This socket consisted of medial-reduced crosslinked polyethylene with a circumferential flange and concentric center (FL-R socket) (Fig. 3). On the other hand, the Charnley offset-bore acetabular cup had eccentric centers [16]. The socket was sterilized using gamma radiation of 25 kGy in nitrogen. Femoral stems were cemented in 32 patients (poor bone quality or aged ≥ 70 years) or fixed without bone cement in 70 patients (good bone quality or aged < 70 years) with good bone quality (reverse hybrid THA). All prosthetic heads were made of 22-mm diameter ceramic (zirconia or alumina) and were attached to the stem with a taper lock. The outer diameter of the acetabular components used in the current study was 40, 42, 44, 46, 48, and 50 mm for 2, 6, 20, 56, 13, and five hips, respectively. The prostheses with an alumina head were implanted with a straight, collarless Titan-6Aluminum-4Vanadium (Ti-6Al-4V) femoral component (cemented or uncemented); those with a zirconia head were implanted using a cemented straight, collarless cobalt-chromium (Co-Cr) femoral component. All prostheses were manufactured by Kyocera Medical Corporation, formerly named Japan Medical Materials Corporation (September 2004 and March 2012, Osaka, Japan).

### Surgical Technique and Postoperative Care

The surgical objective for these hip arthroplasties was to maximize bone coverage by preserving/increasing the acetabulum bone stock using bone grafting techniques that avoided medial protrusion and a high hip center. All procedures were performed with the patient in the lateral decubitus position. A posterolateral approach was used for exposing the hip without osteotomy of the greater trochanter, except in two cases. The medial curtain osteophyte was removed and the dissection proceeded inferiorly to visualize the acetabular fossa, which was the landmark used to start the preparation of the acetabulum. Soft tissue release was important for hip reduction. If necessary, superolateral/circumferential capsulectomy and iliopsoas/subcutaneous adductor tenotomy were performed.

**Fig. 4A–B:**
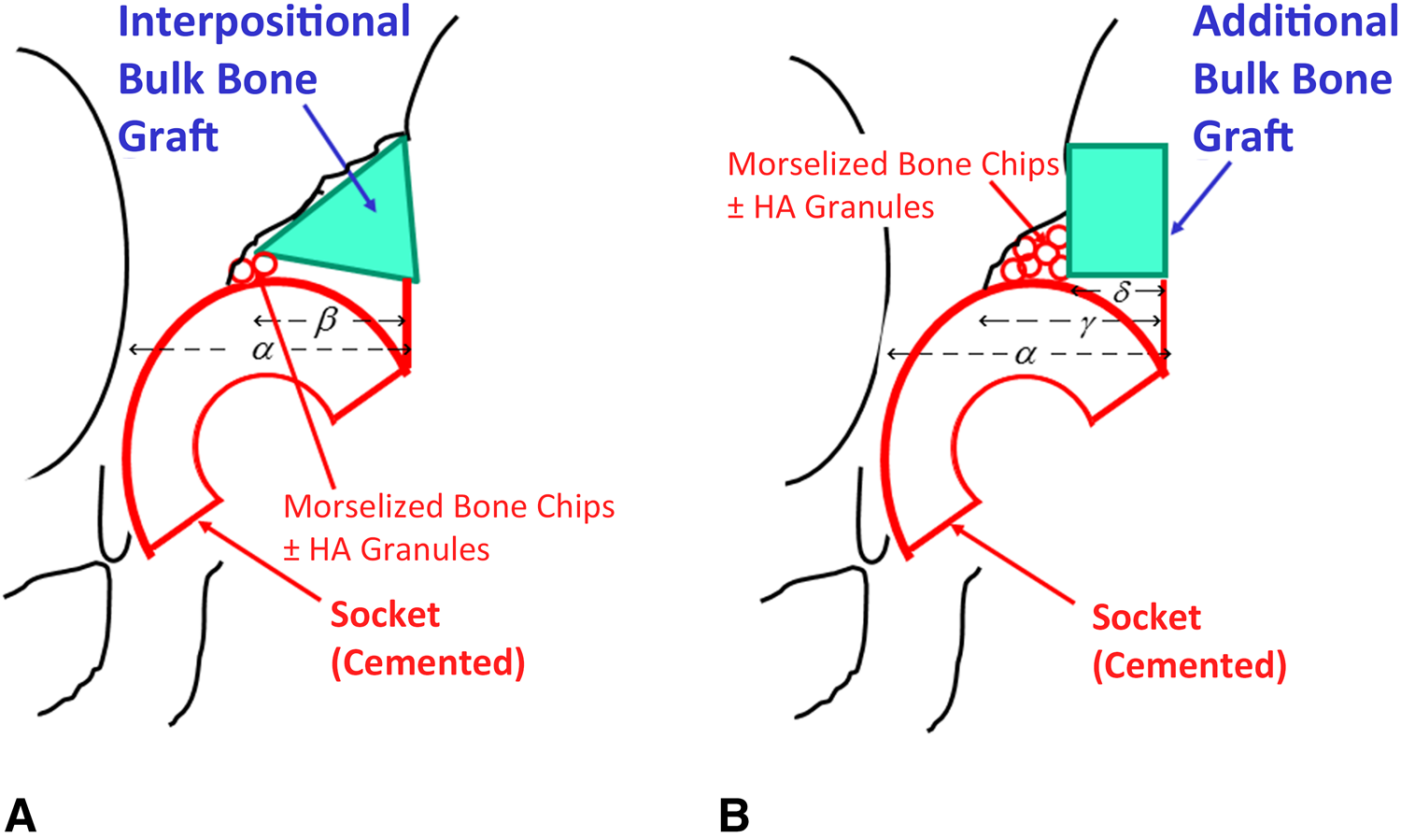
In the interpositional bulk bone grafting method, the coverage ratio of the bulk bone (100 × *β/α* %) was often 50% or more (**A**). On the other hand, in the additional bulk bone grafting method with impaction morselized bone chips (IMBCs), the coverage ratio of the bulk bone (100 × *δ*/*α* %) could be less than 50% (< 50%), whereas the coverage ratio of the bulk bone plus impaction bone grafting (100 × *γ* /*α* %) was > 50% (**B**). *HA* hydroxyapatite.

**Fig. 5:**
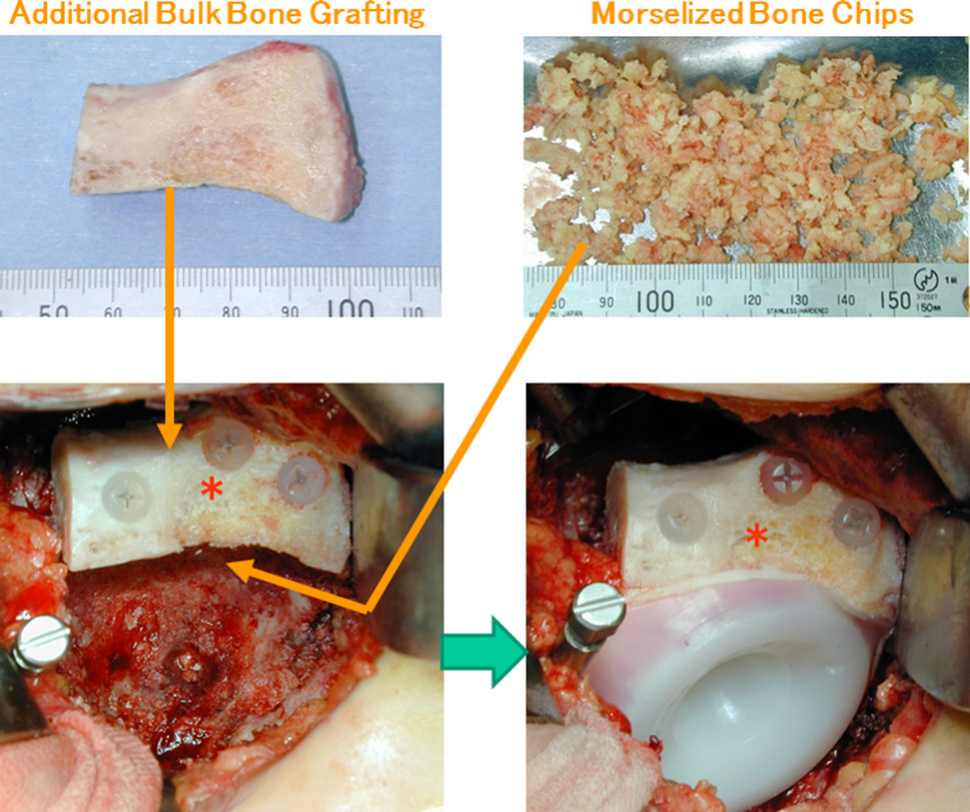
Surgical technique of the additional bulk bone grafting with morselized bone chips for shallow acetabulum is demonstrated. The asterisk represents the additional bulk bone grafting, which is fixed by using poly-l-lactate absorbable screws.

Our goals for reconstruction of shallow and/or steep acetabulum included the avoidance of a high hip center and of excessive medialization of the socket with resulting bone removal. Typically, the additional bulk and impaction morselized bone grafting technique was used to address acetabular roof bone deficiency when the coverage ratio was > 50% and for shallow and/or steep acetabuli with severe bone deficiency such as Crowe Type II, III, or IV [7]. After superolateral or circumferential capsulectomy, for achieving good graft containment, the bulk bone derived from the patient’s resected femoral head was fundamentally placed on the lateral cortex of the ilium and fixed by poly-L-lactate absorbable screws [5] (manufactured by Gunze Co Ltd, Medical Division, Tokyo, Japan, or Takiron Co Ltd, Osaka, Japan) to secure the lateral acetabular roof deficiency located proximally to the acetabular rim (Fig. 4). This was followed by impaction grafting of morselized cancellous bone chips. The morselized bone chips were 2 to 5 mm in diameter (Fig. 5); if the volume of the chips was insufficient, they were augmented using a mixture of porous hydroxyapatite granules (1–6 mm in diameter) with 42% porosity. An all-polyethylene FL-R socket was then fixed with polymethylmethacrylate bone cement. Simplex-P Bone Cement (Stryker, Kalamazoo, MI, USA) was used for the initial 30 hips, and Endurance Bone Cement (DePuy CMW, Blackpool, UK) was used for the remainder.

The bone graft coverage ratio in THA was ≥ 50% in 40% of the additional bulk bone grafting plus impaction morselized bone grafting (40 of 102); the ratio of only additional bulk bone grafting without including the morselized bone was < 50% in all 102 hips.

On the third postoperative day, the patients were allowed to use a wheelchair with touchdown weightbearing after passive and assisted active ROM exercises as instructed by our physical therapists. Crutch use for ambulation was initiated on the 10th to the 14th postoperative day and progressive weightbearing permitted as tolerated. Time to full weightbearing was 3 to 4 weeks postoperatively.

### Followup and Review

After THA, the patients in this study were examined semiannually in the outpatient clinic. From each patient, we obtained a set of AP and lateral (Lauenstein) digital radiographs. One AP radiograph was obtained while the patient was lying supine and another was obtained while standing with full weightbearing on the replaced hip [24]. The radiographs were taken 3 weeks, 2 months, and 6 months postoperatively and during each semiannual followup.

### Radiographic Assessments

All two-dimensional radiographic measurements were recorded by a single researcher (MM) using a computerized measurement system and were evaluated semiannually. A digitizer linked to a computerized radiographic measuring system was used for all measurements.

The extent of acetabular component covered by structural bulk bone and morselized bone grafting (coverage ratio of the bone grafting) was measured on the postoperative radiograph as a percentage of the cup hemisphere (Fig. 4) [20, 40]. Abduction of the acetabular component was measured as the angle formed by the horizontal interteardrop reference line and a line drawn across the face of the cup [20]. The graft material incorporation was evaluated in a time-dependent pattern [9]. According to criteria reported by Knight et al. [20], we also evaluated bridging, graft remodeling, and trabeculation including rounding off of the protruding edge of a graft beyond the socket and the reorientation of the trabecular pattern within the graft to match the normal trabecular orientation of the acetabular roof. Change in graft density in unstressed areas was considered a revascularization indicator [10]. Graft collapse or resorption or breakage of screws used to secure the graft was an indicator of graft failure [20].

Loosening seen on the radiograph was defined as the presence of a continuous (100%) radiolucent zone at the bone–cement interface, wherein the width increased progressively or during changes of position, ie, migration or subsidence of the prosthesis. Migration of the socket seen on the radiograph was defined as either the presence of a ≥ 2-mm position change or rotation. Position changes of the stem seen on the radiograph were defined as the presence of a progressive subsidence of ≥ 2 mm or change of position, eg, varus or valgus [24].

## Results

### Bone Graft Incorporation Over Time

**Fig. 6A–E:**
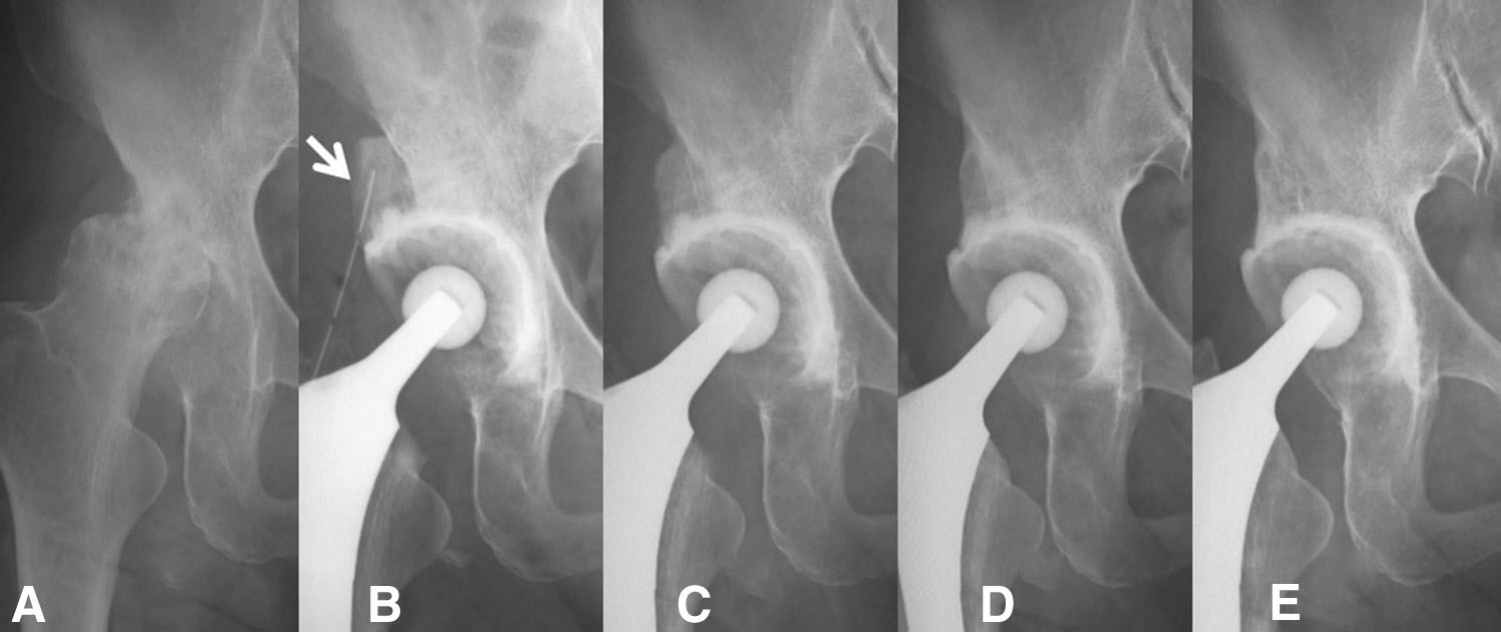
Remodeling and reorientation of the additional bulk bone grafting (arrow) with morselized bone chips for shallow acetabulum are shown. Consecutive radiographs of the left hip of a woman (age at surgery 67 years): preoperatively (**A**), just after THA (**B**), bridging trabeculation observed at 6 months (**C**), remodeling at 12 months (**D**), and reorientation at 36 months postoperatively (**E**).

**Fig. 7A–C:**
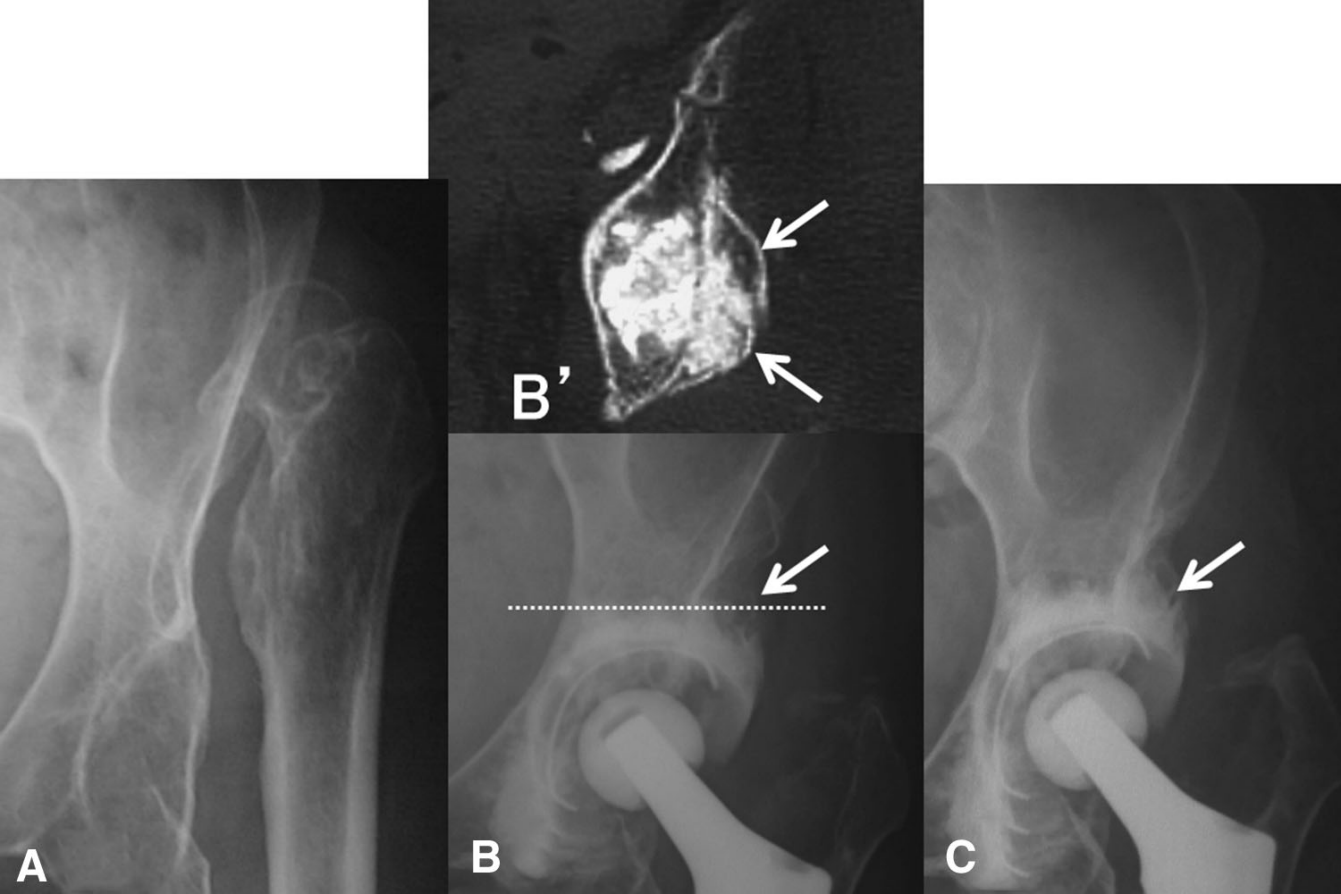
Dislocation case (left hip) with shallow acetabulum. Remodeling and reorientation of the additional bulk bone grafting (arrow) with morselized bone chips plus hydroxyapatite granules are shown in consecutive radiographs of a left hip preoperatively (**A**), 3 weeks (**B**) with CT scanned at the dotted line (**B**′), and 6 years (**C**) postoperatively of a 70-year-old woman at the time of surgery.

For 102 hips with acetabular bone graft reconstruction, prosthetic sockets were rigidly fixed with full incorporation of both the bulk bone graft and the impacted autologous morselized bone; in addition, no cases showed radiographic evidence of resorption of the impacted bone graft over 6 to 15 years of followup. Radiolucent zones between the bulk bone graft and the acetabular roof diminished over time, and in every case, stable fixation of the graft was maintained. We observed that additional structural bulk bone grafting exhibited a time-dependent incorporation pattern. On radiography, bridging trabeculation [20] was observed at 5 ± 2 (mean ± SD) months (range, 2–12 months); remodeling [20] was seen at 9 ± 3 months (range, 2–30 months); and reorientation [20] was noted at 16 ± 5 months (range, 6–42 months) postoperatively. Within 3 years 6 months postoperatively, 101 of 102 additional bulk bone grafting cases showed successful bone remodeling and bone graft reorientation without collapse on radiographs. Both the bulk bone graft and the morselized bone grafts used in acetabular reconstruction in THA functioned well, even over 10 years postoperatively in the additional bulk bone grafting cases. In those cases, the bulk bone graft and the socket remained rigidly fixed with remodeling and reorientation (Fig. 6), even including a hip with Crowe Type IV dysplasia (Fig. 7). In the current series with additional bulk bone grafting, partial resorption of the graft on the lateral side was observed in two cases with socket abduction angles of < 35°. There was neither collapse nor displacement of bulk bone graft at the time of final followup, except in one case with socket loosening. In addition, there was no loosening of the femoral component observed in this group.

### Complications and Clinical Results

In our study, the postoperative Trendelenburg sign was negative except in one hip; in this case, abductor muscles were atrophic and extremely weak at final followup. One postoperative dislocation (1%) was observed and was repositioned manually without revision surgery. One patient experienced transient partial sciatic nerve palsy; complete recovery was observed at 1-year followup. One case presented with deep vein thrombosis of the affected extremity, but no clinical symptoms of pulmonary embolism were found.

Acetabular component in one hip with Crowe Type IV dysplasia showed definite radiographic evidence of loosening and was revised at 9 years postoperatively. In 132 hips (control) without acetabular bone grafting, no loosening was detected. Clinically, the mean Japanese Orthopaedic Association (JOA) score for the 102 hips treated with THA in this study improved from 39 ± 10 points (range, 12–62 points) preoperatively to 95 ± 5 points (range, 72–100 points) postoperatively (p < 0.05, paired t-test) (Table 1). The mean Merle d’Aubigne and Postel score for the hips included in this study improved from 7 ± 2 points (range, 2–12 points) preoperatively to 17 ± 1 points (range, 12–18 points) postoperatively (p < 0.05, paired t-test) (Table 1).

## Discussion

Acetabular bone deficiency, especially lateral acetabular bone deficiency such as shallow dysplasia, is a difficult technical problem associated with primary THA in patients with DDH. Medialization of the acetabular cup to the medial acetabular wall has been described as a routine technique for THA by Charnley [6] and Müller [28]. A benefit of cup medialization in THA has been described as an increased abductor moment arm with a respective increase in the femoral offset [39]. We developed the FL-R socket with the goal of decreasing medial thickness to restore medial bone stock without causing eccentric rotational torque.

The current study evaluated a selected set of patients in a high-volume center, and our results may not be applicable in other settings. Differences in patient size, age, activity level, and ethnicity may also result in different outcomes. We note that patients with dysplasia represent a much larger proportion of patients going on to THA in Japan and other Asian countries than in Europe or North America, and this may also affect the reproducibility of our technique. We did not have a strict control group with whom to compare our results, and it is therefore possible that our patients might have achieved similar outcomes with other, more commonly used techniques. Although we used generally accepted radiographic criteria for bone graft incorporation, we did not use axial or high-resolution imaging, which might provide a more detailed view of bone structure, and we have no histologic or biopsy data to confirm or refute our radiographic findings. Finally, we note that anatomic variability in dysplastic hips is high, and we recommend having other treatment modalities at hand if initial stability cannot be achieved.

We found that this combination of surgical approach (additional bulk bone with impacted morselized bone grafting) and a medial-reduced cemented socket with a concentric center (FL-R socket) resulted in reliable radiographic incorporation of bone graft. Interpositional bulk bone grafting derived from an autologous femoral head for false acetabulum is the most popular method [14, 18, 21, 26, 34, 38] when integrated into the host bone, provides mechanical support to the socket, and is important for long-term success, except for those grafts occupying a large weightbearing area (> 50% coverage) and which eventually collapse [21, 34, 38]. The lower the position of the socket, the greater the extent of the coverage by bone grafting on the acetabular component. This may cause an increase in the late failure rate as a result of collapse of the graft [14, 21, 26, 34]. On the other hand, a satisfactory midterm followup result has been reported with autologous impaction morselized bone grafting in THA with DDH [13, 15, 23]. The sizes of the morselized bone chips were relatively small (2–5 mm diameter) in our study compared with those (7–10 mm diameter) used in revision THA [32] or reconstruction of acetabular protrusion in primary THA [36]. In the current study, the sizes of the bone chips were similar to those (2–4 mm diameter) used in impaction grafting for femoral bone deficiency of revision THA [11].

The large majority of patients treated with this combination of approach and implant installed in the original acetabulum achieved durable implant fixation at a mean of 10 years. In shallow or steep acetabuli, the hip center is located proximally and laterally with superolateral bone deficiency. An elevated hip center is often used for reconstructing steep acetabuli without bone grafting but often results in unacceptable lateral displacement of the hip [17, 31]. Furthermore, loss of bone stock, abductor muscle weakness, increased bone impingement, and an increased rate of loosening have been observed in cases with a high hip center [29, 37]. Therefore, we do not recommend superior placement of an implanted socket for acetabular reconstruction. For acetabular reconstruction in THA, it is important to lower and medialize the high hip center into the original acetabulum.

Patients in this series achieved consistent improvements in terms of pain and function as measured by the JOA and Merle d’Aubigne scores without collapse of the bone graft. The grafted bone functioned well for over 10 years, although partial resorption of the additional bone graft on the lateral side was observed in two hips (2%). Complications such as transient partial sciatic nerve palsy, deep vein thrombosis without pulmonary embolism, and dislocation were not serious and had a low rate of incidence (1%). These clinical outcomes and complications were comparable to those of cemented [21] or cementless THA [18] with bulk femoral head autograft. We recommend that the current reconstruction methods using a medial-reduced socket and additional bulk bone with impacted morselized bone grafting might be applied for cemented socket fixation because it might be difficult to press-fit a cementless metal shell to grafted bulk and morselized bone. However, the shell with screw fixation might be included in the current reconstruction methods.

The goal of this study was to accomplish stable reconstruction not only for perpendicular, but also for horizontal bone deficiency of the acetabular roof by implanting a medial-reduced polyethylene socket and additional bulk bone with impacted morselized bone grafting. Grafted bone remodeling with healing and a good clinical outcome were achieved by evaluation of radiographic examination as well as according to JOA and Merle d’Aubigne and Postel scores in midterm followup. Longer term outcomes should be the subject of further investigation.
